# Active offset-frequency control of optical frequency comb via sum-frequency mixing of passively mode-locked laser and continuous-wave laser

**DOI:** 10.1038/s41598-024-63627-2

**Published:** 2024-06-12

**Authors:** Hansol Jeong, Deok Woo Kim, Hyunhak Kim, Myoungsik Cha, Han Seb Moon

**Affiliations:** 1 Department of Physics, Pusan National University, Geumjeong-gu, Busan, 46241 South Korea; 2 Quantum Sensors Research Center, Pusan National University, Geumjeong-gu, Busan, 46241 South Korea

**Keywords:** Atomic and molecular physics, Optical physics

## Abstract

We propose a method for actively controlling the frequency of an optical frequency comb (OFC) using sum-frequency generation (SFG) with a nonlinear crystal. For the first time, OFC generation was experimentally demonstrated via sum-frequency mixing of a narrowband continuous wave (CW) laser and a passively mode-locked fiber laser. By adjusting the optical frequency of the CW laser, we successfully controlled the offset-frequency of the SFG-OFC, which was mapped from the OFC of the pulse pump laser. Furthermore, by comparing the spectral widths of the SFG-OFC modes generated from two CW lasers with different spectral widths, we confirmed that the spectral characteristics of the SFG-OFC modes depended on the spectral features of the CW laser.

## Introduction

Optical frequency combs (OFCs) have revolutionized a wide range of scientific and technological fields, including optical frequency standards, high-resolution spectroscopy, and multi-dimensional quantum states^1–16^. Characterized by a set of equally spaced optical frequencies, the OFC serves as a powerful tool for achieving precise frequency measurements and the generation of coherent light sources. However, in many applications, the active control of the frequencies of the OFC modes is essential.

The frequency spacing of an OFC is determined by the repetition rate of the mode-locked pulsed laser used in the setup, and active control of the repetition rate is critical^15,16^. Traditionally, controlling the frequency modes of an OFC using mode-locked fiber lasers required precise control of the resonator length of the laser, which can be expensive and complex^4–21^. On the other hand, the offset-frequency of the OFC is normally adjusted by manipulating the carrier-envelope offset phase of the pulsed laser^1–3^. Particularly, controlling the carrier-envelope offset frequency of a mode-locked pulsed laser often involves using intricate devices to manipulate the laser frequency^17–19^. Precise control of the OFCs is particularly important for high-resolution spectroscopy applications^9–15^.

However, these limitations have prompted the exploration of alternative approaches; we have previously demonstrated an OFC based on an acousto-optic modulator (AOM) operated in an actively mode-locked pulse mode, utilizing the injection-seeding technique^13^. The repetition rate was controlled using an AOM. Another OFC based on stimulated Brillouin scattering was demonstrated in a periodically poled lithium niobate (PPLN) waveguide^22^. However, the experimental demonstration of the active control of optical frequency comb generation using sum-frequency mixing of passively mode-locked laser and continuous-wave laser has thus far not been reported.

In this study, we report a sum-frequency generation (SFG) process using a narrowband CW laser and a passively mode-locked laser and present an alternative method for active offset-frequency control of an OFC generation via SFG. Our approach leverages the principles of SFG with a nonlinear crystal to achieve precise control over the OFC modes. By exploiting the SFG process in a nonlinear crystal, we experimentally demonstrate the active frequency control of the SFG-OFC. Specifically, we employ a narrowband CW external cavity diode laser (ECDL) as the reference source for frequency control. The ECDL beam is collinearly combined with a passively mode-locked laser beam in a nonlinear crystal to generate the OFC via SFG.

By adjusting the optical frequency of the ECDL, the frequency offset of the SFG-OFC generated through the SFG process can be successfully controlled via adding the optical frequency of the ECDL to that of the OFC of the mode-locked laser. This active control mechanism allows an OFC with a tunable center frequency which could be changed for specific application requirements. Furthermore, we investigate the spectral characteristics of the OFC modes by comparing the results obtained using two CW lasers with different spectral widths. This comparative analysis clearly demonstrates the dependence of the SFG-OFC mode features on the properties of the CW laser employed as one of the two input fields of the SFG. Our proposed scheme offers a promising method for applications in various fields such as photon-pair generation from atomic system and two-photon spectroscopy using OFCs.

## Experimental schematic

**Figure 1:**
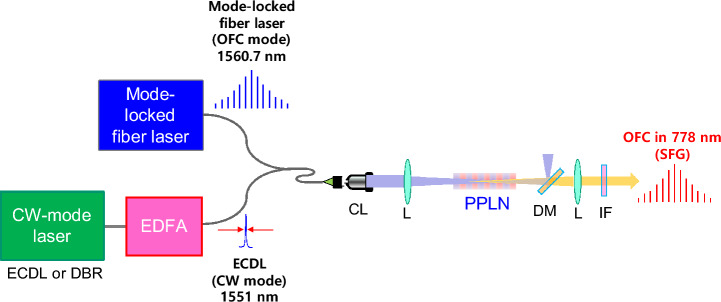
Experimental schematic for sum-frequency generation (SFG) in a PPLN crystal utilizing a narrowband continuous-wave external cavity diode laser (ECDL) and a passively mode-locked fiber laser (DBR: distributed Bragg reflector laser; *EDFA* erbium-doped fiber amplifier, *CL* collimation lens, *L* lens, *DM* dichroic mirror, *IF* interference filter.

The experimental schematic for the proposed method for active frequency control of an OFC using SFG in a nonlinear crystal is depicted in Fig. 1. In this experiment, we employed a mode-locked fiber laser and an ECDL as the fundamental input sources for the SFG process. The mode-locked pulsed fiber laser featured a center wavelength of 1560.7 nm, repetition rate of 28.26 MHz, pulse width of approximately 1.3 ps, spectral width of approximately 1.1 nm, and average output power of 2.4 mW. The ECDL had a center wavelength of 1551.2 nm with a linewidth of approximately 1 MHz. The power of the ECDL incident on a PPLN crystal was set to 150 mW via amplification with an erbium-doped fiber amplifier.

To maximize the spatial overlap between the two input laser beams, the beams were combined using a fiber coupler. The spatial mode distribution of the combined beams was refined by passing them through a single-mode fiber. Both beams were linearly polarized parallel to the z-axis of the PPLN crystal using fiber polarization controllers. After the combined beams were collimated in free space, they were focused on the PPLN crystal using a lens (L) with a focal length of 400 mm. The 1/e^2^ intensity radius of the beam waist was measured to be 69 μm using the knife-edge scanning method. In this case, the Rayleigh range was estimated to be 19 mm, which was approximately twice the length of the PPLN crystal (10 mm). The period of the domain inversion was Λ = 19.4 μm for type-0 quasi-phase-matched (QPM) SFG. A dichroic mirror (DM) was incorporated to eliminate the pump light in the 1550 nm band, allowing the measurement of the SFG signal in the 778 nm band with an interference filter. At the measurement site, we employed a power meter, avalanche photodiode, and spectrometer to measure the spectral features. In particular, our proposed method offers the capability to actively control the frequency offset of the SFG-OFC via the continuous tuning of the ECDL. The optical frequency of our SFG-OFC can be scanned by adjusting the optical frequency of the ECDL. The center wavelength of our SFG-OFC is corresponding to the 778-nm for the two-photon resonance of the 5S_1/2_–5P_3/2_–5D_5/2_ transition of Rb atom^23^. Furthermore, the bandwidth of SFG-OFC is enough narrow for avoiding the 780-nm for the single-photon resonance of the 5S_1/2_–5P_3/2_ transition of Rb atoms. We believe that the two-photon resonance with the SFG-OFC is applied to generate the pulse-mode operating photon pair via spontaneous four-wave mixing process from cascade-type atomic systems via the 5S_1/2_–5D_5/2_ transition of ^87^Rb atom^24–27^.

## Optical frequency comb via sum-frequency generation

**Figure 2:**
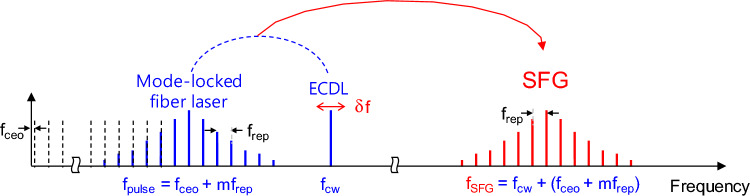
Sum-frequency-generated optical frequency comb ($f_{SFG}$) from the mode-locked fiber pulse ($f_{pulse}$) and the CW-ECDL lasers ($f_{cw}$).

The SFG maps the frequency comb of the mode-locked laser in the spectral regime of approximately 1561 nm to that in the regime of approximately 778 nm, as illustrated in Fig. 2. The mode-locked pulse laser generates a regularly spaced pulse sequence in the time domain, which is equivalent to an OFC in the frequency domain. The OFC of the mode-locked fiber laser has two degrees of freedom. The optical frequency of the *m*th tooth of a self-referenced comb of a mode-locked fiber laser can be described as $f_{pulse} = mf_{rep} + f_{ceo}$, where the uniform frequency spacing $f_{rep}$ is equal to the pulse repetition rate of the mode-locked laser, and the initial offset frequency $f_{ceo}$ (< $f_{rep}$) is related to the carrier-envelope phase offset in the time domain. Sum-frequency mixing of the pulsed and CW lasers (frequency: $f_{cw}$) produces new OFC frequencies that are simply given by the sum

1 $$ f_{SFG} = f_{pluse} + f_{cw} \, = mf_{rep} + f_{ceo} + f_{cw} . $$

However, we also need to consider the simultaneously occurring second-harmonic generation (SHG) of the pulse laser because its output power can be significantly greater than that of the SFG, even though it is not quasi-phase-matched. The QPM conditions were calculated using Sellmeier’s formula for a 5 mol% MgO-doped congruent lithium niobate crystal^28^. The QPM temperature of the SFG of the input waves at 1560.7 nm (center wavelength of the mode-locked laser) and 1551.2 nm (CW laser) was estimated to be 70.7 °C, while that of the pulse SHG was 106 °C. The temperature bandwidths were estimated as 9.3 °C for SFG and 8.5 °C for SHG, assuring sufficient discrimination between the two nonlinear interactions in terms of the crystal temperature. SHG of the CW laser can be neglected because its band is too narrow to overlap with the SFG band.

**Figure 3:**
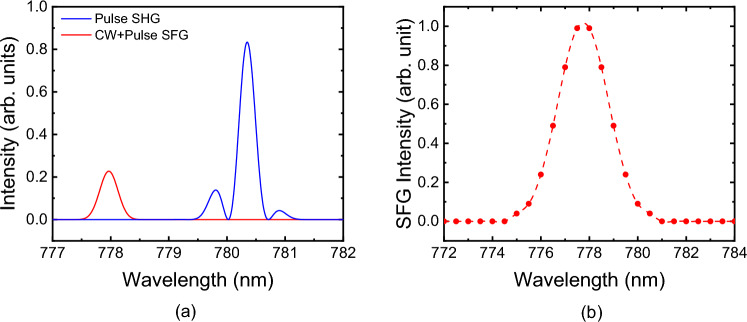
(**a**) Calculated output power spectra of SFG (red) and SHG (blue) at 70.7 °C. (**b**) Measured power spectrum of SFG at 75.0 °C.

We calculated the SFG and SHG outputs assuming negligible depletion of the fundamentals, obtaining

2 $$ I_{SF} (\lambda_{SF} ) = C\left[ {\frac{{d_{eff} L}}{{\lambda_{SF} n_{SF} }}{\text{sinc}} \left( {\frac{{\Delta k_{SF} L}}{2}} \right)} \right]^{2} I_{CW} I_{Pulse} , $$

3 $$ I_{SH} (\lambda_{SH} ) = C\left[ {\frac{{d_{eff} L}}{{\lambda_{SH} n_{SH} }}{\text{sinc}} \left( {\frac{{\Delta k_{SH} L}}{2}} \right)} \right]^{2} I_{Pulse}^{2} , $$

where the subscripts *SF* and *SH* indicate that the physical quantity is for the SFG and SHG processes, respectively. Here, *I* represents the intensity of the participating waves, *L* the interaction length (= 10 mm), *n* the refractive index, and *d*_eff_ the effective nonlinear coefficient, which is common to the two processes. In addition, *C* denotes a common constant and $\Delta k$ the wave-vector mismatch (including the QPM grating wave-vector), which depends on the wavelengths of the participating waves and the crystal temperature. The intensities $I_{CW}$ and $I_{Pulse}$ are assumed to have Gaussian spectral distributions with widths of 0.8 × 10^−6^ nm and 1.1 nm, respectively. If slight differences in the wavelength and refractive index can be neglected in the two equations, the relative output intensities can be obtained by considering only ${\text{sinc}}^{2} \left( {\frac{1}{2}\Delta k_{SF} L} \right)I_{CW} I_{Pulse}$ and ${\text{sinc}}^{2} \left( {\frac{1}{2}\Delta k_{SH} L} \right)I_{Pulse}^{2}$ for SFG and SHG, respectively. Using the dispersion relation given in Ref.^28^, Eqs. (2) and (3) can be plotted under the QPM condition for the SFG (70.7 °C), as illustrated in Fig. 3a. The SFG band is centered at 778 nm, whereas the center of the SHG band is at 780.4 nm. The QPM bandwidths of both interactions were estimated as 0.60 nm in terms of the output waves. Although non-QPM SHG is stronger than QPM SFG because the peak intensity of the pulse input is approximately 440 times greater than that of the CW input, the SFG band is well separated from the SHG band at this temperature.

In our SFG experiment, the PPLN crystal temperature was set to 75.0 °C to maximize the SFG-OFC. This slight difference from the theoretically calculated QPM temperature of 70.7 °C is partially attributed to the difference between the actual temperature of the beam path in the PPLN crystal and that of the external measuring point. The output SFG was separated from the SHG using the interference filter (IF; Semrock Inc.; LL01-780-12.5) and tunable filter (Photon etc. Inc.; LLTF VIS-2). A dichroic mirror (DM; Semrock Inc.; BLP01-1064R-25) was incorporated to eliminate the pump light in the 1550 nm band. The measured SFG spectrum in Fig. 3b appears to be approximately three times broader than the calculated QPM bandwidth of 0.6 nm, due to the limited resolution (~ 2 nm) of the tunable filter. The average output power of the SFG was measured as approximately 0.15 μW. The power increase of the SFG-OFC is important for various applications. When the powers of mode-locked pulse laser and CW laser are increased, we can consider the relatively high-power of the SFG-OFC.

### Active control offset-frequency of OFC

**Figure 4:**
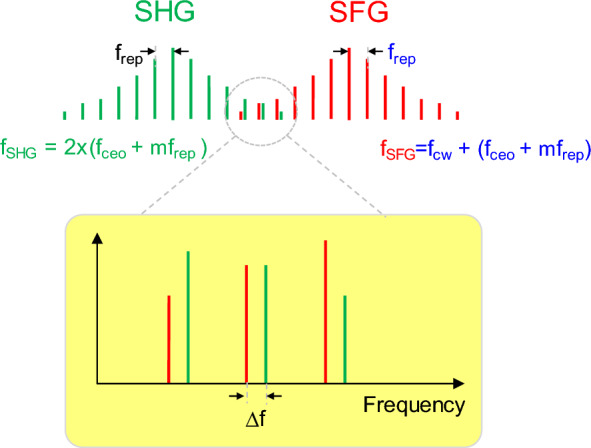
Diagram showing the overlap between the SHG-OFC (green lines) and SFG-OFC (red lines). The bottom inset shows the magnified overlap region, where Δf represents the frequency difference between the nearest modes of the SFG-OFC and SHG-OFC.

To verify the efficacy of our active frequency control method for the SFG-OFC, we investigated the beat signals between the SHG-OFC and SFG-OFC in relation to the optical frequency of the ECDL. We note here that the spectral characteristics of the mode-locked fiber laser used in our experiment deviate from the ideal Gaussian spectrum. Although the two spectra appear to be completely separated in Fig. 3, the two OFC tails may overlap partially, as shown schematically depicted in Fig. 4. Consequently, it is possible to observe the beat signals between the SHG-OFC (green lines) and SFG-OFC (red lines), which were achieved in our experiment by adjusting the QPM conditions.

While the frequencies of the SFG-OFC are given by Eq. (1), because of the identical mode-locked fiber laser ($f_{pulse} = mf_{rep} + f_{ceo}$), those of the SHG-OFC are expressed as

4 $$ f_{SHG} = 2f_{pluse} \, = 2mf_{rep} + 2f_{ceo} . $$

The intermode beat frequencies between the two OFCs can be obtained by subtracting Eq. (4) from Eq. (1):

5 $$ \Delta f_{m} = \left| {f_{SHG} - f_{SFG} } \right| = \left| {mf_{rep} + f_{ceo} - f_{cw} } \right|. $$

These beat frequencies correspond to the difference between the frequency ($f_{cw}$) of the CW ECDL and those of the modes of the mode-locked fiber laser. Among them, a few of the slowest beats, corresponding to modes whose frequencies are close to $f_{cw}$, can be easily observed with an RF spectrometer.

**Figure 5:**
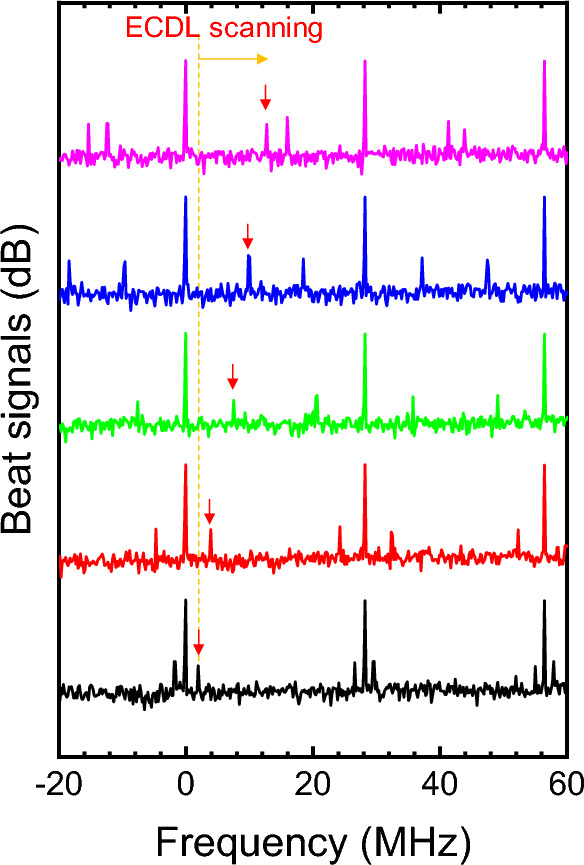
RF spectrum of the beat signals according to the optical frequency tuning of ECDL (orange arrow). Red vertical arrows indicate the slowest intermode beating.

Figure 5 shows the RF spectrum of the beating signals resulting from the overlap in the spectral regime around 779 nm, under the experimental condition of a QPM temperature of 90.0 °C. In addition to the intermode beat signals, the stronger intramode beat frequencies (corresponding to the repetition rate of the mode-locked fiber laser, 28.26 MHz and its multiples) were also observed. These intramode beat signals are greater than the intermode beat signals by ~ 30 dB because the output of SHG is considerably stronger than that of SFG at this temperature. When the CW-laser input was blocked, the intermode beat signals disappeared, leaving only SHG intramode beat signals.

To confirm the role of the CW-laser input in the SFG-OFC, we finely adjusted the optical frequency of the ECDL within the range of 10 MHz and observed significant changes in the beat signals of the OFC generated through the SFG, as indicated by the red arrows in Fig. 5. While the frequency offset of a conventional OFC is adjusted by manipulating the carrier-envelope offset phase of a mode-locked pulse laser, our proposed method offers the capability to actively control the frequency offset of the OFC via the continuous tuning of the ECDL. Although the adaptive dual-comb spectroscopy has been used the beat note of a free-running optical comb line and a stabilized Hz-linewidth CW laser^29^, we experimentally demonstrated the SFG process using a MHz-linewidth ECDL and a passively mode-locked fiber laser. We tried to answer about the following questions. First, is it possible to achieve SFG process between CW and pulse lasers? Second, what is the properties of SFG? Third, can be used for an alternative method for active offset-frequency control of an OFC generation via SFG? This SFG-OFC opens up possibilities for OFC-based applications in fields such as two-photon spectroscopy. However, the optical frequency of the SFG-OFC can be stabilized to reference the absolute frequency used for the two-photon Rubidium transition of ^87^Rb, even in the presence of drift in either the offset frequency or the repetition rate of the passive mode-locked pump laser. Only the offset frequency of the SFG-OFC can be stabilized using the CW laser. In fact, to fully stabilize the comb in our SFG-OFC scheme, repetition rate locking remains necessary.

**Figure 6:**
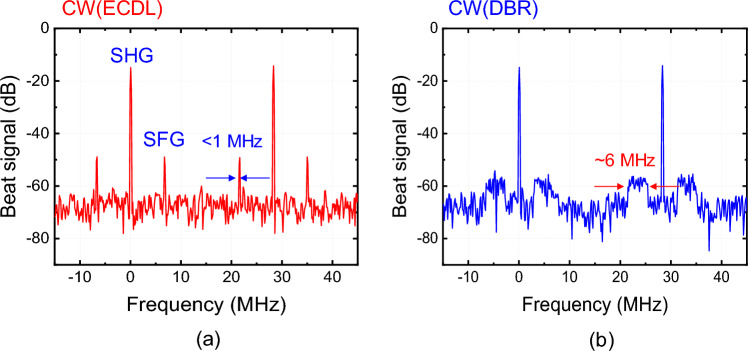
Beat spectra of SHG and SFG-OFCs with two different CW lasers; (**a**) an ECDL with a linewidth of < 1 MHz and (**b**) a distributed Bragg reflector (DBR) laser with a linewidth of approximately 6 MHz.

To investigate the influence of the CW laser on the SFG-OFC, we compared the beat signals for two different CW lasers: an ECDL with a linewidth of < 1 MHz and a distributed Bragg reflector (DBR) laser with linewidth of approximately 6 MHz. The beat signals in each case are shown in Fig. 6a, b. Although the SHG beat signals remained constant, the characteristics of both SFG beat signals were influenced by the linewidths of the CW and pulse pumps. These results show that the properties of the OFC depend on the optical frequency of the CW pump. Furthermore, comparing with the signal to noise ratio (SNR) of beat signal in the two conditions, the SNR with the ECDL is approximately 5 dB higher than that in the DBR laser.

## Conclusion

In conclusion, we successfully demonstrated the offset-frequency control of an OFC using SFG in a PPLN crystal. This control was achieved by integrating a narrowband continuous wave (CW) laser and a passively mode-locked laser. Precise tuning of the optical frequency of the ECDL enabled effective manipulation of the narrowband OFC modes generated via the SFG process in the PPLN crystal. This approach presents a novel means for enhancing the capabilities of OFC-based technologies. Overall, our proposed method holds promise for realizing OFC-based applications in fields such as two-photon spectroscopy and photon-pair generation from atomic ensemble.

## Data Availability

The datasets used or analyzed during the current study available from the corresponding author on reasonable request.
